# The SMC1-SMC3 cohesin heterodimer structures DNA through supercoiling-dependent loop formation

**DOI:** 10.1093/nar/gkt303

**Published:** 2013-04-24

**Authors:** Mingxuan Sun, Tatsuya Nishino, John F. Marko

**Affiliations:** ^1^Department of Molecular Biosciences, Northwestern University, Evanston, IL 60208-3500, USA, ^2^Department of Molecular Genetics, National Institute of Genetics, The Graduate University for Advanced Studies, 1111 Yata, Mishima, Shizuoka 411-8540, Japan and ^3^Department of Physics and Astronomy, Northwestern University, Evanston, IL 60208-3118, USA

## Abstract

Cohesin plays a critical role in sister chromatid cohesion, double-stranded DNA break repair and regulation of gene expression. However, the mechanism of how cohesin directly interacts with DNA remains unclear. We report single-molecule experiments analyzing the interaction of the budding yeast cohesin Structural Maintenance of Chromosome (SMC)1-SMC3 heterodimer with naked double-helix DNA. The cohesin heterodimer is able to compact DNA molecules against applied forces of 0.45 pN, via a series of extension steps of a well-defined size ≈130 nm. This reaction does not require ATP, but is dependent on DNA supercoiling: DNA with positive torsional stress is compacted more quickly than negatively supercoiled or nicked DNAs. Un-nicked torsionally relaxed DNA is a poor substrate for the compaction reaction. Experiments with mutant proteins indicate that the dimerization hinge region is crucial to the folding reaction. We conclude that the SMC1-SMC3 heterodimer is able to restructure the DNA double helix into a series of loops, with a preference for positive writhe.

## INTRODUCTION

Structural Maintenance of Chromosomes (SMC) complexes are present in eukaryotes, prokaryotes and archaea, and are responsible for a variety of chromosome-organizing functions (1–3). In general, SMCs are thought to act as linkers between chromatin segments. Eukaryote condensin SMCs play a key role in mitotic chromosome condensation (4), while eukaryote cohesin SMCs hold sister chromatids together during mitosis until they are specifically degraded during anaphase (5–7).

Apart from its function of building sister chromatid cohesion, cohesin also plays an essential role in double-stranded DNA break repair (8,9) and gene regulation, and mutations in cohesins and their regulators are associated with numerous human developmental diseases (10). Gene-regulatory functions of cohesin follow from its involvement in maintenance of interphase chromatin ‘loop’ organization (11,12). Yeast cohesins have been observed to mediate interactions *in cis* along individual chromatids (13), and metazoan cohesin SMCs are known to mediate looping interactions in the interphase nucleus, guided in a subset of cases by interactions with the domain boundary factor CTCF (12) or mediator complexes (11). However, despite it being established that cohesin plays a central role in chromatin organization, exactly how cohesins physically interact with chromosomal DNA is still an open question.

All SMC complexes are built around a heterodimeric core containing two large (>1000 aa) proteins, each of which consists of, in sequence, a globular ‘hinge’ domain, a long (50 nm) coiled-coil region, and a globular ATP-binding ‘head’ domain. The core proteins dimerize *via* the hinge domains, which are thought to permit appreciable conformational flexibility of the coiled-coil arms. The head domains are also able to dimerize if they have bound ATP, suggesting that SMCs function might involve cycling between an ‘open’ and a ‘closed’ state, controlled in part by ATP binding and hydrolysis (14).

Both condensin, which is based on the SMC2-SMC4 core heterodimer, and cohesin, based on a SMC1-SMC3 core (below, SMC1/3), are found *in vivo* associated with a third ‘kleisin’ (CAP-H for condensin and Scc1 for cohesin), which links the two heads to form a trimeric protein ring. Experiments in yeast have established that cohesin trimers are capable of topologically encircling DNA double helices (15,16). This topological linking function has been proposed to be the basis of sister chromatid cohesion, a possibility supported by electron microscopy observation of a rather open cohesin ‘ring’ structure. Yeast condensin trimers have also been found to topologically encircle DNA (17), which is intriguing given condensin’s less-open structure observed *via* electron microscopy (18).

**Figure 1.**
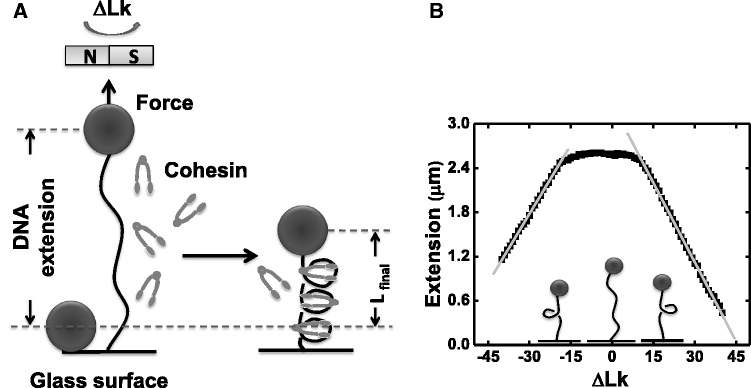
Experimental design, calibration of DNA extension versus ΔLk (**A**) An 9.8 kb DNA linearized pFos-1 is attached at one end to glass cover slip, the other to a 2.8 µm magnetic bead (filled circles). Upon protein flow through, the DNA length change owing to cohesin/DNA interaction can be detected. (**B**) Calibration data for DNA extension as a function of linking number change (ΔLk) under 0.45 pN force, in buffer with no protein present. At zero supercoiling, the tether extends to ∼2.7 µm. For <12 turns, there is little or no change in DNA extension. Beyond 12 turns, the DNA molecule buckles and plectonemic regions are generated, resulting in a simple linear reduction of DNA extension with ΔLk (for ΔLk > 11, slope is −62.3 ± 0.4 nm). For ΔLk < −14 turn, molecule buckles and the slope of the linear plectonemic regions is nearly the same as that of positive supercoiling. (for ΔLk < −14, slope is 71.2 ± 0.5 nm).

Single-DNA micromechanics experiments (Figure 1) can monitor changes in extension resulting from protein binding for DNAs under defined tension and supercoiling, and provide an approach well-suited to study of the physical interaction of SMC complexes with DNA. Prior single-molecule experiments on condensin pentamers (SMC2-SMC4 heterodimer plus the kleisin plus two additional subunits) purified from *Xenopus laevis* eggs observed condensin to mediate a DNA compaction reaction, which proceeds in roughly 70 nm (∼200 bp) steps (19). This reaction was observed to absolutely require ATP, but to not have a strong dependence on DNA supercoiling.

Here we carry out single-molecule experiments on the core cohesin heterodimer purified from budding yeast, with the objective of determining how the SMC1/3 complex interacts with DNA. We show that SMC1/3 can drive stepwise DNA compaction that does not require ATP and is DNA-supercoiling dependent. Our results establish that SMC1/3 can stabilize interactions between distinct DNA segments with preferences for specific DNA topologies, and have broad implications for cohesin’s role in chromosome organization.

## MATERIALS AND METHODS

### Proteins, DNA constructs and buffers

Cohesin SMC proteins and kleisin subunits were purified and prepared as described (20,21). Most experiments were carried out on DNA derived from linearized pFOS-1 (9.6 kb, 3.3 μm contour length), and were end-labeled by ligation of 900 bp polymerase chain reaction fragments multiply labeled by digoxigenin and biotin; the multiple labels allow the molecules to be torsionally constrained. A small number of experiments were carried out using 48.5 kb λ-DNA (Promega), which was end-labeled by ligation of single-stranded DNA oligos carrying one biotin or one digoxigenin. These tethers are able to swivel around their end-connections and are never torsionally constrained. All experiments were carried out at 30°C in 20 mM Tris–HCl, pH 7.5 buffer with 100 mM NaCl added.

### DNA tethering and magnetic tweezers experiments

Single-molecule experiments were performed by attaching the DNA constructs to streptavidin-coated paramagnetic particles (M-280, Dynal), and to the inside of an anti-digoxigenin-functionalized glass flow cell (Figure 1A). Flow cells were mounted in a magnetic tweezers setup (22), which allowed tethered particles to be rotated and pulled by controlled forces while tracking their 3D positions. In brief, force applied on DNA was controlled by movable permanent magnets; DNA linking number is set by rotating the magnets and therefore the magnetic field; the orientation of the bead is fixed by the magnetic field direction. The flow cell design allows the solution surrounding the tethered DNAs to be changed in a few seconds.

We monitored bead position, and therefore DNA extension, using digital image analysis, using a lab-built microscope based on a 100× 1.3 NA (Olympus) microscope objective. The vertical position of the objective was controlled using a piezoelectric positioner (Piezojena). A digital camera (A741 CCD camera, Pixelink) was used to acquire data to a PC. Bead tracking was done using a lab-written bead tracking software (Labview, National Instruments), which measured the coordinates of the tethered bead and a nearby bead attached to the flow cell surface. Bead position in the image plane (xy) was tracked using centroid analysis. Bead position normal to the imaging plane (z) was measured using Fourier analysis of the bead images via comparison with calibration data obtained for images of the bead using a range of focal plane positions before the experiment. Measurement of the difference between tethered and fixed bead positions allows correction for instrumental drift. Data were collected at ∼60 Hz. The results for this method of z-position measurement were validated for each experiment using a slower autofocus method to measure the distance between tethered bead and surface bead for a series of DNA extensions.

For single-DNA experiments, before protein was introduced, it was checked that the tethered bead was attached by one DNA molecule, *via* a series of DNA extension and corresponding force measurements at different magnet positions. The force on the bead was measured using bead fluctuations in the xy plane as described previously (22). In short, mean-squared variance of the bead position in the plane is inversely proportional to applied force. Measurement of extension and force at ∼5 magnet positions provides a test that a single molecule is tethering the bead, using the known elastic response of DNA (22) and the known tether length. Molecules that did not match the expected elastic response were not studied. The force-extension calibration data also provide data for conversion of magnet position to applied force.

Following force-extension calibration of the 10 kb molecules, they were put under the tension desired (0.45 pN for most experiments) and tested for torsional stiffness by rotating the magnets. For torsionally constrained molecules, sufficient rotation leads to reduction of extension; beyond a well-defined ‘buckling’ point, the extension of the DNA changes linearly with linking number (Figure 1B) as plectonemic supercoils are formed, while DNA torque remains nearly constant (23,24). Measurement of extension versus ΔLk also allowed determination of the ΔLk = 0 point from the maximum extension (generally the initial value of Lk) and verification of the stability of stored linking number. The experiment control program keeps track of magnet rotations and therefore ΔLk, which can be changed to any value as required during an experiment. Molecules that showed no change in extension with rotation were used for experiments on ‘nicked’ torsionally unconstrained DNA.

Following characterization of the tethered DNA, protein solution was introduced into the flow cell, allowing measurement of the extension of protein–DNA complexes.

## RESULTS

### SMC1/3 generates stepwise DNA compaction in the absence of ATP

**Figure 2.**
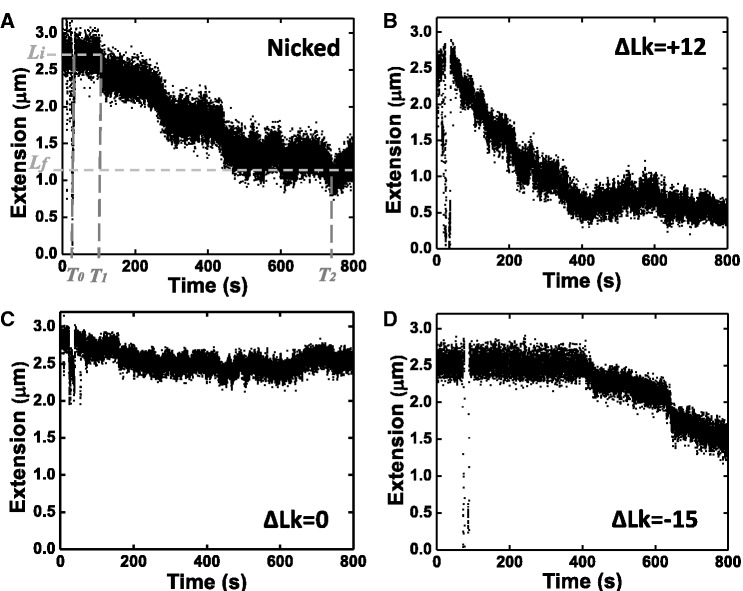
Sample traces for DNA compaction observed in various topological states. (**A**) Nicked DNA. (**B**) Positively supercoiled DNA (ΔLk = +12). (**C**) Un-nicked DNA with ΔLk = 0. (**D**) Negatively supercoiled DNA (ΔLk = −15).

We found that SMC1/3 can drive a stepwise DNA compaction reaction on nicked (torsionally unconstrained) DNA. Figure 2A shows a typical time course for compaction against a constant 0.45 ± 0.05 pN force, following addition of 10 nM SMC1/3 in 100 mM NaCl reaction buffer without ATP to the flow cell (introduction of enzyme occurs at time t = 0 in all time courses). A series of step decreases (and occasional increases) in extension were observed. The compaction is highly force-dependent: 1 pN completely stalls the reaction, with no folding steps (Supplementary Figure S1A). For forces <0.45 pN, faster compaction occurs, but with a final DNA extension of approximately the same as in the 0.45 pN case (Supplementary Figure S1C). The force dependence and the stepwise nature of the compaction reaction are both indicative of loop formation.

The compaction reactions were reversible in the sense that following them, replacement of SMC1/3 solution with protein-free buffer led to a gradual release of DNA extension on an ∼10–20 min time scale (Supplementary Figure S1D). This is consistent with the observation that in addition to forward steps (Supplementary Figure S1E), reverse steps also occur (Supplementary Figures S1E and S4), indicating microscopic reversibility of the compaction steps.

Subsequent experiments were carried out at 0.45 pN. This force is optimal for a few reasons. First, as mentioned above, higher forces eliminated the compaction reaction, while lower forces lead to higher amplitude and lower frequency of tethered bead motion (Brownian noise) and less DNA extension, reducing vertical bead position resolution. Second, in experiments where ΔLk is constrained, ∼0.5 pN force leads to a torque (DNA torsional stress) at buckling and during formation of plectonemic DNA comparable with the torque in a supercoiled DNA with |σ| ∼ 0.05 (23,25,26), and comparable with torques likely to be found *in vivo*. Prior single-DNA experiments on the *Escherichia coli* SMC MukB and on *Xenopus* condensin I have focused on forces ∼0.5 pN for similar reasons.

### DNA compaction by SMC1/3 is stimulated by positive torsional stress

Rotation of the beads and therefore linking number of un-nicked DNA can also be controlled, by rotation of the magnets. Figure 1B shows extension as a function of linking number (ΔLk) for a single naked DNA molecule under 0.45 ± 0.05 pN force in protein-free reaction buffer. As DNA is overwound, plectonemic regions start to form when ΔLk = +11. As linking number is increased beyond ΔLk = +11, plectonemic supercoils form, causing DNA extension to decline nearly linearly with ΔLk, by −72 ± 1 nm/ΔLk. For underwinding beyond ΔLk = −14 turns, DNA extension decreases linearly by 62 ± 1 nm/ΔLk.

A series of experiments were carried out for tethers held at 0.45 pN with the following DNA topological states: nicked DNA, un-nicked but torsionally relaxed DNA (ΔLk = 0), slightly positively supercoiled DNA (ΔLk = +12) and slightly negatively supercoiled DNA (ΔLk = −15). These positive and negative linking number values correspond to one turn past the ‘buckling’ point where plectonemic supercoils just start to form for the ∼10 kb DNA held at 0.45 pN tension. In each case, 10 nM SMC1/3 solution without ATP was added at time t = 0.

We note that DNA torque rises from zero roughly linearly as ΔLk is changed from zero, but then is nearly constant while plectonemic supercoiling is being generated. For 0.45 pN and 100 mM salt, DNA torque is ∼6 pN^.^nm at buckling and during plectoneme formation (23,25,26). During plectoneme formation at 0.45 pN, the superhelical density in the plectonemic region is approximately |σ| = 0.05 (23). For comparison, the linking numbers of the molecule as a whole at buckling are lower, 0.0125 at ΔLk = +12 and 0.0156 at ΔLk = −15. The added linking number tends to be partitioned to the plectonemic region because of its higher writhe and lower free energy at a given superhelical density (23,27,28).

Steady stepwise DNA compaction occurred following addition of SMC1/3 for both nicked and positively supercoiled (ΔLk = +12) DNA molecules, usually proceeding until the final DNA extension was approximately one-third of its initial length (Figure 2A and B). DNA compaction was relatively slow on negatively supercoiled DNA molecules (ΔLk = −15), with long pauses between steps (Figure 2D), but eventually SMC1/3 was able to compact DNA to roughly the same degree as in the positive supercoiling case. For un-nicked torsionally relaxed DNA (ΔLk = 0), while step events did occur, the folding reaction was extremely slow (Figure 2C) and did not reach the same degree of compaction over a waiting time of 2 h.

**Figure 3.**
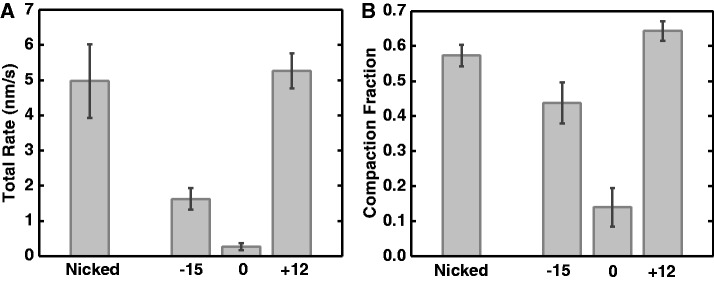
Supercoiling dependence of DNA folding reaction by cohesin SMC1/3. (**A**) Total compaction rate. Positively supercoiled and nicked DNAs had the fastest rates of folding. Negatively supercoiled DNA was compacted more slowly, while un-nicked DNA with ΔLk = 0 was folded only very slowly. (**B**) Total compaction fraction showed a similar dependence on DNA topological state; note un-supercoiled un-nicked DNA was only slightly compacted. For each group of DNA, the number of experiments *n* = 15, 19, 15 and 15 for nicked DNA, ΔLk = −15, 0 and +12 DNA, respectively.

Data from a series of experiments for each of these topological states (nicked, ΔLk = −15, 0, +12, all at 0.45 pN tension) were quantified by measuring the initial (naked DNA) extension *L_i_* and the final extension *L_f_*. *L_f_* was determined from the nearly flat tail regions of the time series (not shown in truncated time series of Figure 2; see Supplementary Figure S1F for a tail region, and Supplementary Figure S1G for the sample trace of a complete reaction). This determined the extension change (Figure 2A, *L_i_−L_f_*) and the time interval between addition of protein and when the final length was reached (Figure 2A, *T_2_−T_0_*). The resulting total rate (*L_i_−L_f_*)/(*T_2_−T_0_*) and compaction fraction (*L_i_−L_f_*)/*L_i_* averaged over a series of trials are shown in Figure 3, which shows that the fastest, most robust reactions were for ΔLk = +12 DNA. Positively supercoiled and nicked DNAs are favorable substrates for compaction by SMC1/3, relative to un-nicked molecules with either negative or zero supercoiling.

The slow rates and low compaction fractions for ΔLk = 0 DNA in part reflect that in ∼75% of those experiments there was essentially no compaction at all (Supplementary Figure S2A). But even within the subset of experiments for which a compaction reaction occurred, the same pattern of differences in compaction fraction (Supplementary Figure S2B), lag time between protein addition and the beginning of the compaction (*T_1_−T_0_*, Supplementary Figure S2C), and compaction rate following the lag time ([*L_i_−L_f_*]/[*T_2_−T_0_*], Supplementary Figure S2D) were observed (Supplementary Information). We conclude that positive supercoiling stimulates DNA compaction by SMC1/3.

Lastly, as positive supercoiling accelerated the DNA-folding reaction, we examined the effect of varied levels of positive supercoiling (+5, +12, +20 and +30). Our results indicate that the SMC1/3 compaction reaction is driven primarily by DNA torsional stress, which increases steadily before plectonemes start to form at ΔLk = +11 (26), rather than by plectonemic interwinding because during plectoneme formation DNA torque is nearly constant [(23–26), see Supplementary Information and Supplementary Figure S3].

### SMC1/3 compaction proceeds via ≈130 nm steps

For all DNA topological states, the compaction reactions were observed to proceed *via* stepwise extension changes (Figure 2). Histograms of step sizes for ΔLk = +12, −15, 0 and for nicked molecules (Supplementary Figure S4) were all similar, with a large peak with mean 130 ± 10 nm. Reverse steps were observed, indicating the microscopic reversibility of the compaction reaction, and are shown in Supplementary Figure S4 as negative step sizes; the reverse steps are of nearly the same size as the forward steps. The step size distributions indicate that the compaction involves similar reorganizations for different topological states of DNA.

### Concentration dependence of binding suggests that the compaction reaction is not strongly cooperative

**Figure 4.**
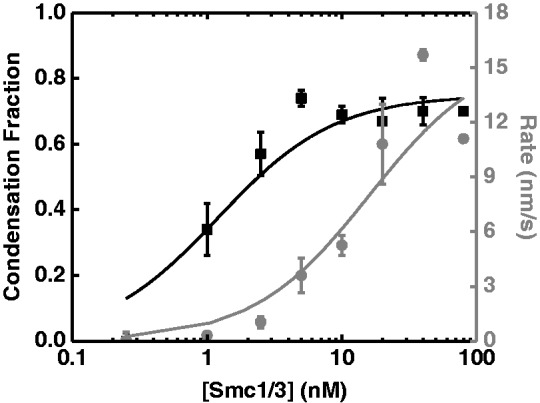
SMC1/3 concentration dependence of the compaction fraction and reaction rate for ΔLk = +12 DNA molecules. The equilibrium compaction fraction (black squares) was fit into Hill equation {θ = [SMC1/3]^n^/([K_a_]^n ^+ [SMC1/3]^n^)} with a Hill coefficient *n* = 0.97 ± 0.16 and a K_a_ = 1.2 ± 0.5 nM (black curve). The reaction rates (gray circles) were also fit into a Hill equation, with a Hill coefficient *n*_r_=1.20 ± 0.24, K_r_ = 12 ± 3.9 nM (gray curve).

We next examined the SMC1/3 concentration dependence of the fraction of compaction and reaction rate, for ΔLk = +12 DNAs. The final (equilibrium) compaction fraction (Figure 4, black) becomes smaller at low concentrations, indicating a concentration dependence of the number of SMC1/3 heterodimers bound in a DNA-condensing mode. This curve fits a Hill form {θ = [SMC1/3]^n^/([K_a_]^n ^+ [SMC1/3]^n^)} with Hill coefficient *n* = 0.97 ± 0.16 and a K_a_ = 1.2 ± 0.5 nM, suggesting non-cooperative binding of SMC1/3 heterodimers to DNA. The compaction rate also fits to the Hill form (Figure 4, gray, *n*_r_ = 1.2 ± 0.2, K_r_ = 18 ± 4 nM); the value of *n* ≈ 1 again indicates that the binding kinetics are not highly cooperative, consistent with the result for the compaction fraction.

It may appear surprising that the reaction should be non-cooperative given the slow rate for its completion. However, for the ΔLk = +12 reactions that we analyzed the concentration dependence of in detail, the average lag time was ∼40 ± 10 s (Supplementary Figure S2C); by comparison, assuming an average step size of 130 ± 10 nm (Supplementary Figure S4B) and an average compaction rate of 5.2 ± 0.5 nm/s (Supplementary Figure S2D), the average time between steps is 25 ± 3 s. Thus the lag time is only slightly longer than what would be expected for independent binding, also consistent with the compaction reaction being weakly cooperative. The approach to well-defined final states during which reverse as well as forward steps occur (Figure 2 and Supplementary Figure S4) is consistent with a largely non-cooperative reaction. The reaction is slow largely because the individual step events occur at a slow rate. However, we do caution that the crude Hill analysis used in Figure 4 assumes an ‘all or none’ cooperative binding model, and serves mainly to indicate that the compaction reactions do not show a strong concentration dependence clearly indicative of strong cooperativity.

### Compaction fraction saturates at ∼70%

The compaction fraction saturates at ∼0.7 (Figure 4), beyond ≈5 nM SMC1/3 concentration, in experiments on the 10 kb DNA with ΔLk = +12. We carried out experiments on 48.5 kb (16.5 µm) λ-DNA tethers (22) with entirely different sequence and of nicked topology (with 10 nM SMC1/3), which showed nearly the same compaction fraction as experiments with nicked 10 kb molecules (Supplementary Figure S5). Thus, there is a limited amount of compaction that can be driven by SMC1/3. This is distinct from strongly DNA-condensing proteins such as *Bacillus subtilis* RacA, which is capable of tightly folding DNA, pulling magnetic particles all the way to the tethering surface in similar single-molecule experiments (29). In contrast, reactions with >5 nM SMC1/3 appear to form a structurally defined DNA–protein complex of ∼30% of the original DNA contour length.

### Rapidly changing DNA linking number reorganizes SMC1/3 on DNA

**Figure 5.**
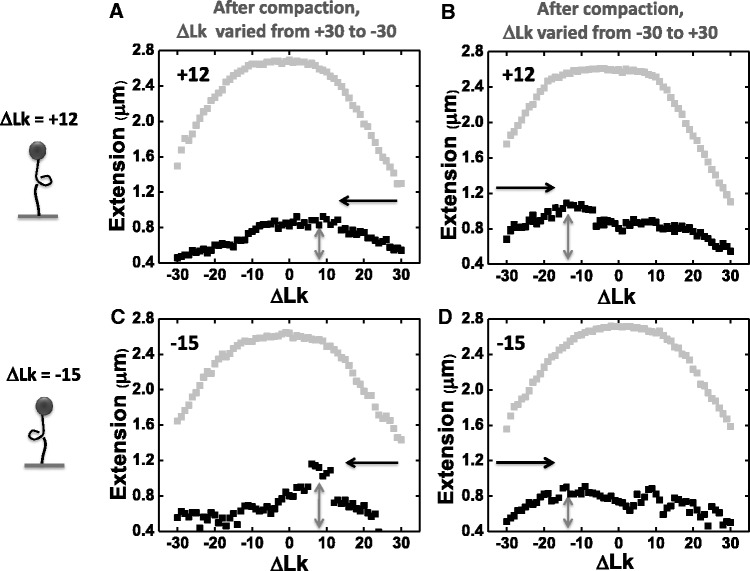
Changing DNA linking number results in SMC1/3 redistribution. Naked DNA at constant force shows a reversible extension versus linking number response centered at ΔLk = 0 (gray squares). However, this extension versus linking number response was altered in the presence of SMC1/3 protein (black squares). (**A** and **B**) experiments with DNA of ΔLk = +12 before protein addition, after reaction reaches equilibrium, DNA extension was measured as a function of ΔLk either from +30 to −30 (A), or from −30 to +30 (B). (**C** and **D**) Experiments with DNA of ΔLk = −15 before protein addition, after reaction reaches equilibrium, DNA extension was measured as a function of ΔLk either from +30 to −30 (C), or from −30 to +30 (D). Analysis of these repeated experiments in each group results in average peak linking number of ΔLk = 6.8 ± 1.2 (*n* = 8), −10.5 ± 2.9 (*n* = 3), 8 ± 1.5 (*n* = 3) and −11.9 ± 1.2 (*n* = 7) turn for A, B, C and D, respectively.

Naked DNA at constant force shows a reversible peaked extension versus linking number response with peak at ΔLk = 0 (Figure 1B; Figure 5, gray squares). In the presence of proteins, one expects to see a shift in the peak of the DNA extension-ΔLk response in a direction indicative of the tendency for the protein to locally change DNA twist or writhe (30). With the objective of analyzing the chirality of the compacted SMC1/3-DNA complex, we carried out experiments where stressed DNAs with either ΔLk = +12 or −15 were first allowed to bind SMC1/3, and then extension was measured as a function of ΔLk. We used relatively rapid cycling of ΔLk (1 s per each turn added) with the objective of measuring the response of the SMC1/3/DNA complex on a time scale shorter than the ∼20 min associated with dissociation of the protein (Supplementary Figure S1D).

For naked DNA, the extension-ΔLk curve is the same regardless of the direction that ΔLk is varied (Figure 5, gray squares). However, after compaction by 10 nM SMC1/3 in solution, we found that the peaks of extension-ΔLk curves (Figure 5, black squares; arrows indicate the direction that ΔLk is varied) were dependent on the direction that ΔLk was changed; i.e. whether following compaction, ΔLk was set first to +30 and then varied to −30 (Figure 5, left column), or alternately set first to −30 and then varied to +30 (Figure 5, right column). The peak location was determined by fitting a quadratic function to the extension-ΔLk data in a linking number range ±10 around the largest extension. Notably, the peak location was approximately the same during these measurements, independent of whether the initial ΔLk during compaction was +12 (Figure 5, top row) or −15 (Figure 5, bottom row). Instead, the peak locations were determined by the direction of the scan following compaction.

We note that relatively little SMC1/3 was able to dissociate in these experiments, as the extension at ΔLk = 0 remained much less than that obtained in the ΔLk = 0 experiments that were allowed to reach binding equilibrium (Figures 2C and 3B). Therefore these experiments are out of SMC1/3 binding equilibrium, further evidence for which is shown by the dependence in peak position on direction of ΔLk scan (Figure 5). These results indicate that the contribution of SMC1/3 to constraint of DNA twist and writhe can be readily altered by varying DNA torsional stress, on a time scale shorter than that required for substantial protein unbinding.

### Deletion of most of the head domains only partially eliminates DNA compaction by SMC1/3

**Figure 6.**
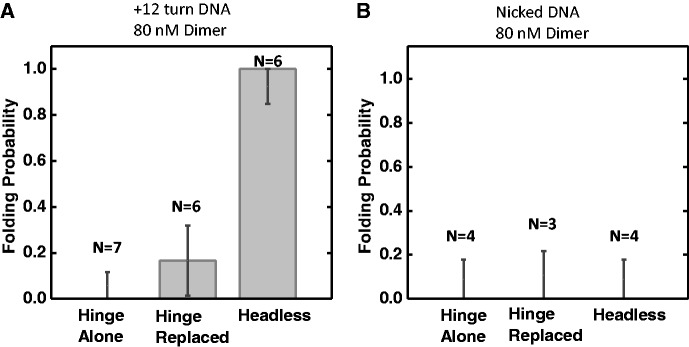
DNA compaction by SMC1/3 mutants. DNA folding probability was plotted for hinge domain alone, hinge replacement mutant and headless mutant at 80 nM SMC1/3 protein concentration on ΔLk = +12 DNA (**A**) or nicked DNA (**B**). The number of experiments done was indicated on each bar. Error bars represent standard error.

To investigate the role of the globular ‘head’ domain in SMC1/3 compaction, which contains the ATP-binding, ATP-hydrolyzing and possible DNA-binding sites (31), we studied reactions with ‘headless’ (HL) mutant heterodimers, in which residues 1–167 and 1074–1225 of SMC1 and residues 1–174 and 1068–1230 of SMC3 (roughly 90% of the head regions) had been deleted. Compared with WT SMC1/3, this HL mutant can still form the coiled-coil arms, but does not have the globular head regions, as shown by AFM imaging (Supplementary Figure S6). This mutant did not condense DNA of any topology at a concentration of 10 nM. However, higher concentration (80 nM) of this mutant was able to drive stepwise DNA compaction on positively supercoiled DNA (Figure 6A and Supplementary Figure S7A, ΔLk = +12), but not on nicked DNA (Figure 6B and Supplementary Figure S7A, nicked). Thus the head deletions suppressed, but did not completely eliminate, the ability of SMC1/3 to condense DNA.

### Replacement of the hinge domains strongly suppresses DNA compaction by SMC1/3

To examine the role of the hinge domain in SMC1/3 compaction, experiments were carried out with mutants for which the hinge region of SMC1 was replaced with mouse p14, while the hinge region of SMC3 was replaced by mouse MP1, which binds the p14 protein. This ‘hinge replacement’ (HR) construct has been previously studied; the MP1/p14 interaction has been shown to successfully mediate dimerization of the SMC1 and SMC3 arms, and is strong enough to resist spindle forces in yeast (21). However, the HR mutant was not able to condense any topology of DNA for concentrations up to 80 nM (Figure 6 and Supplementary Figure S7C and D). Thus the HR mutant has DNA-folding activity suppressed more than the head-deletion mutant, indicating that the hinge region plays a more crucial role in the compaction reaction.

Experiments with the wild-type SMC1/3 hinge domain alone, lacking any of the coiled-coil and head domains, showed no stepwise compaction at 80 nM concentration (Figure 6 and Supplementary Figure S8). The hinge domain is necessary but not sufficient for stepwise compaction of DNA by SMC1/3.

### Separation of DNA binding and loop capture functions using salt concentration

**Figure 7.**
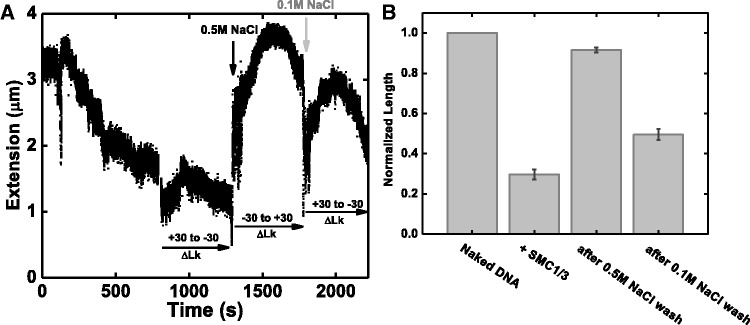
Alternation of DNA binding and looping function by varying salt concentration. (**A**) A 13 kb DNA with ΔLk = +14 was first compacted by 10 nM SMC1/3 under 0.5 pN force (0–800 s); DNA torsional response was tested by scanning the DNA extension from ΔLk = +30 to −30 with protein in solution (800–1300 s). Then we washed out the free protein in solution with protein free buffer containing 0.5 M NaCl; a fast DNA decompaction was observed (black arrow), and DNA torsional response was recovered to appear as naked DNA (1300–1750 s). Finally, we flush the flow cell with reaction buffer containing 0.1 M NaCl, and rapid DNA compaction was observed (gray arrow); DNA torsional response was also altered (1750–2150 s). (**B**) Average normalized DNA extension (extension relative to naked DNA contour length) at different salt concentration. First bar represents naked DNA before protein addition. The second bar represents the final DNA length in the presence of SMC1/3, normalized to naked DNA length; the average is 0.30 ± 0.03. After washing with reaction buffer containing 0.5M NaCl, the average DNA length is 0.92 ± 0.02 (third bar). After changing buffer back to reaction buffer containing 0.1 M NaCl, the average DNA normalized extension decreased to 0.50 ± 0.03 (fourth bar). Averages were computed over five experimental trials.

The mutant SMC1/3 experiments suggested that the compaction reaction involves interactions of distinct sites on the heterodimer with DNA. Hypothesizing that these distinct interactions might be affected differently by elevated salt, we carried out experiments where first, a supercoilable 13 kb DNA under 0.5 pN force was compacted by 10 nM wt SMC1/3 in 100 mM NaCl buffer as in the previous experiments (Figure 7A, times from 0 to 800 s). Then, we washed away the protein solution with protein-free buffer containing 500 mM NaCl; rapid unfolding of the SMC1/3–DNA complex was observed, and the resulting tether had the torsional response of naked DNA (Figure 7A, time 1500–1800 s), showing no sign of SMC1/3 binding. However, when we then flushed 100 mM NaCl protein-free solution through the flow cell, rapid partial refolding occurred (Figure 7A, times after 1800 s), indicating that the protein stayed bound to DNA during the elevated salt wash, and was then able to re-condense DNA after buffer exchange to 100 mM NaCl reaction buffer.

In a series of experiments of this type, we found that the unfolding caused by the 500 mM NaCl buffer returned the SMC1/3-DNA complex to ∼90% of its original naked extension (Figure 7B, third bar). Subsequent reintroduction of 100 mM NaCl protein-free buffer always re-compacts DNA (Figure 7B, fourth bar). These experiments suggest that there are two distinct DNA-binding modes for SMC1/3: DNA binding and DNA looping. The DNA-looping interactions that cause DNA compaction are disrupted by 500 mM NaCl, while the DNA-binding interactions are still present under those elevated-salt conditions.

### *In trans* SMC1/3 interaction between ‘braided’ DNAs

Our experiments with single DNAs indicate that SMC1/3 is able to condense DNA *in cis* by formation of relatively short loops, but do not show whether SMC1/3 can interact with distant DNA sites *in trans*. To determine if this was possible we carried out experiments with beads attached by pairs of nicked DNA molecules, which can be wrapped around one another, or ‘braided’, by rotation of the magnetic field (Supplementary Text and Supplementary Figure S9A). These braid structures can be compacted by 10 nM SMC1/3 in the 100 mM NaCl buffer (Supplementary Figure S9B), but this might be solely due to compaction *in cis* along the two DNAs without *in trans* contacts.

Evidence for *in trans* interactions can be detected by examining the extension as a function of braid linking number (catenation number Ca). Supplementary Figure S9C shows DNA extension at 0.5 pN as a function of catenation number, for a naked DNA braid (top black squares) and for the same braid after binding of 10 nM SMC1/3 (lower gray squares), using 1 rotation/s to cycle from Ca = −1 to +15 and then to −15 is ∼45 s, much less than the binding or unbinding timescale. In the naked DNA case, there is a narrow peak at zero catenation number (Supplementary Figure S9C, black squares) corresponding to establishment of the first crossing by half a turn (32,33) (Supplementary Figure S9A, left-most two pictures). After compaction by SMC1/3, this feature is absent (Supplementary Figure S9C, gray squares), indicating that the two DNA molecules are attached to one another by at least one SMC1/3 ‘bridge’ (Supplementary Figure S9A, right-most sketch). We note that while the compaction fraction of single DNA tethers was one-third of the initial DNA length (Figure 3B and Supplementary Figure S5), the double-DNA tether was folded almost completely (Supplementary Figure S9B), suggesting that there are more SMC1/3-DNA contacts than would occur for *in cis* compaction of two parallel DNA molecules.

## DISCUSSION

Cohesin’s *in vivo* functions include sister chromatid cohesion, long-range tethering of promoter to enhancer DNA in interphase chromatin and a role in DNA double-strand break repair. All of these functions require cohesin to act to stabilize interactions between disjoint DNA segments. Cohesin–DNA interactions are known to be influenced by loading factors (Scc2-Scc4) (34,35), transcription factors (e.g. mediator complex or CTCF) (11,36) and the DNA double-strand break repair machinery (9). Here we have shown that the core heterodimer alone (SMC1/3) is able to directly interact with DNA to drive a stepwise compaction process, and to mediate interactions between separate DNA molecules. Using mutant versions of the heterodimer we have also shown that the hinge region of SMC1/3 plays a major role in the heterodimer’s interaction with DNA.

### Stepwise DNA compaction by SMC1/3 heterodimer

We observed that 10 nM SMC1/3 is able to compact DNA molecules against 0.5 pN forces via a stepwise reaction, with individual steps of well-defined size of ∼130 nm. The stepwise reaction and its force dependence are strongly indicative of loop formation as the basis for the compaction. This compaction reaction stops after about two-thirds of the length of the molecule is incorporated into folded form, even with excess SMC1/3 in solution, suggesting a well-defined structure of the DNA–SMC1/3 complex rather than an indiscriminately cohesin-cross-linked nucleoprotein ‘globule’. Our observations are most consistent with a complex consisting of a linear ‘tandem’ series of DNA loops, each containing roughly 130 nm (400 bp) of DNA, or about three persistence lengths of DNA. Based on the compaction fraction, the spacing of the loop bases should be roughly half this distance, or ∼70 nm (200 bp). Therefore, we estimate that there are ∼10–20 cohesin-mediated DNA loops on a 10 kb DNA molecule in our experiments.

### SMC1/3-driven DNA compaction is dependent on DNA torsional stress

Prior biochemical studies of cohesin-DNA interactions indicate a binding preference for supercoiled DNA relative to linear DNA, but without a clear conclusion regarding a preferred chirality (31,37,38). Our study establishes that the core cohesin heterodimer has a preference for compaction of positively supercoiled DNA substrates. The DNA compaction reaction is sensitive to DNA torsional stress, with positive torsional stress increasing the rate and degree of the compaction reaction over experiments with negative torsional stress or on ΔLk = 0 substrates.

These results can be qualitatively explained by a ‘tandem loop’ picture, given that the heterodimer has a preference for positive-writhe (right-handed) loops. For positive torsional stress (ΔLk = +12), formation of positive-writhe loops is accelerated, facilitating the compaction reaction. For an equivalent amount of negative torsional stress (ΔLk = −15), the reaction is slower, and slightly less complete, indicating less-favorable SMC1/3 binding to negative-writhe loops. For ΔLk = 0, there is no torque driving formation of loops with either sign of writhe, and formation of the more favorable positive-writhe loops faces a ‘twist energy blockade’ caused by the constraint of Lk sufficient to stop the reaction from proceeding. By contrast, for nicked DNA, positive-node loops may form with no twist energy cost, and compaction can go forward, although less vigorously than in the ΔLk = +12 case.

Cohesin’s preference for DNA under positive torsional stress may facilitate targeting cohesin to pre-replicated DNA. During DNA replication, positive torsional stress is generated upstream of replication forks. As the replication forks progress, right-handed sister-chromatid intertwining (SCI) is likely to be found in replicated DNA, providing another suitable binding substrate for cohesins. A recent study shows that SCI persists after S-phase in a cohesin-dependent manner (39). SCI aid with sister chromosome cohesion, but might be prematurely resolved by topo II before anaphase. Cohesin may act to stabilize SCIs, and to act against spindle pulling forces to strengthen sister chromatid cohesion until cohesin proteolysis occurs. A similar preference for positively writhe DNA has been observed for bacterial condensin MukB, *Xenopus* condensin I, and yeast SMC2/4, in bulk biochemical assays (40–42), suggesting a conserved preference for positively writhed DNA.

Intriguingly, yeast centromeres are highly enriched in cohesin (13,43), and there is evidence suggesting that positive supercoiling is induced by centromeric chromatin (44,45). It is conceivable that the preference for positive supercoiling we have observed helps to target cohesin to yeast centromeres. The arrayed loop formation we have observed may be related to cylindrical arrays of cohesin observed on pericentric chromatin during yeast mitosis (13,43,46).

Notably, cohesin exhibits different interactions with linear un-nicked DNA (ΔLk = 0) and nicked DNA: compaction was largely suppressed on ΔLk = 0 DNA substrates, but not on nicked DNA. This result can be explained by the energy cost of buildup of torsional stress by chiral loops, which does not occur for nicked DNA. A simple explanation for all these effects is a preference of cohesin to bind to DNA crossings, with a preference for positive-writhe loops when they are on the same DNA molecule. Furthermore, SMC1/3 is able to reorganize dynamically on DNA in response to torsional stress (Figure 5). For tandem loops, this could be accomplished by switching the chirality of individual loops.

### Topology dependence and prior experiments

Losada and Hirano compared binding of human (HeLa) cohesin complexes with same-length DNAs of different topology, with the result that negatively supercoiled plasmids bound ∼3-fold more cohesin than did nicked circles (37). Cohesin bound linear DNA poorly by comparison, and positively supercoiled DNA was not studied. While this may appear different from our results, there are a number of differences between the experiments. First, Losada and Hirano used complete cohesin complexes, whereas we have studied the core heterodimer SMC1/3 from yeast. Second, Losada and Hirano measured binding to molecules under zero tension with and without complete plectonemic supercoiling, while we measure compaction of DNA under mild tension and torque, largely without plectonemic supercoiling. Third, the starting conformation of the DNAs studied by Losada and Hirano can be expected to be different in that their plectonemically supercoiled DNAs are rather compact and have many near crossings, the nicked circles are less compacted and have fewer crossings, and the linear DNAs are still less compacted and have still fewer crossings (47). In summary, the results of Losada and Hirano indicate that cohesin will bind more readily to DNA molecules which have more self-crossings, and that there is no strong effect of ATP on the binding reaction.

In our experiments, the mild (0.45 pN) tension and relatively low level of supercoiling (+12 or −15 turns on a 10 kb plasmid) maintains all the molecules in an initially nearly fully extended conformation, but with the possibility of crossing formation by thermal fluctuation (48,49). Our experiments compare SMC1/3’s ability to trap loops along DNA in a way that is relatively unbiased by overall DNA geometry: our main result is that positive-writhe loops are more readily trapped than negative-writhe loops. Loops along a ΔLk = 0 molecule are more difficult to trap, presumably due to the higher free energy cost of negative-writhe loops needed to counterbalance positive writhe, so as to keep ΔTw and the resulting twist free energy from growing too large. Our main result, that SMC1/3 captures DNA loops in an ATP-independent manner, indicates SMC1/3’s affinity for DNA crossings, and is in accord with the results of Losada and Hirano.

Our readout of compaction likely cannot detect SMC1/3 bound to plectonemic regions of a supercoiled molecule because those regions are already compacted. Hence we have focused most of our attention on DNA without extensive plectonemic supercoiling. However, one might expect to find additional SMC1/3 bound to crossings in plectonemic regions, which might be detected using labeled SMC1/3 in an experiment that combined magnetic tweezers and fluorescence visualization.

Onn and Koshland showed that in a crude yeast extract, cohesin was assembled more readily onto DNAs immobilized on beads at both their ends (‘looped’), relative to the case where only one end of the DNA was immobilized (‘unlooped’) (50), and that the assembly reaction was ATP-dependent. Dependence of the reaction on DNA torsional stress was not studied. While the biochemical situation in crude extracts is different from that for the purified SMC1/3 studied here, the preference for the looped substrate are again consistent with a preference of cohesin for DNA crossings, which will be less probable in the unlooped case. However, it is possible that the ‘bare ends’ of the unlooped molecules play a role. The high degree of complexity of the extract-based system, which likely assembles chromatin onto DNAs carrying known cohesin binding sites and which contains cohesin ‘loading factors’, make it likely distinct from our assay for SMC1/3 binding to DNA. A challenge for the future is to use single-molecule methods to recapitulate and analyze the reactions found *in vivo—*which we presume to be similar to those occurring in the extract system—using substrates with cohesin binding sites and purified enzymes.

### Roles of different domains of SMC1/SMC3 in DNA compaction

To further investigate the role of each domain in DNA folding by SMC1/3, we studied two functional mutant constructs: the HR SMC1/3 and a HL SMC1/3. The HR construct has been shown to be still able to form the trimetric cohesin ring and hydrolyze ATP *in vitro*, but is not able to associate with chromosomes *in vivo* (20). A later study (21) demonstrated that the positively charged channel of the wt hinge domain is required for cohesin to mediate sister chromatid cohesion. In our study, the HR construct failed to condense DNA at any DNA supercoiling states, even at high protein concentrations (80 nM). However, the hinge by itself is unable to generate DNA folding, indicating that the hinge region is absolutely necessary but not sufficient for DNA compaction.

This leaves the question of what other part of the protein is involved in the stepwise compaction reaction. Given that the ATPase head domains of SMCs have been thought to be involved with DNA binding (51), we were surprised to see that the HL construct lacking almost all of the head domain was still able to drive complete stepwise compaction at 80 nM concentration. Evidently, the ATPase domain is not absolutely required for the stepwise compaction reaction. We propose that the hinge domain makes initial physical contact with DNA, and the SMC arms help with the DNA looping by stabilizing the cross-overs of DNA loops (Supplementary Figure S10). This also explains the strong chiral preference of the DNA looping, due to the asymmetry of the cohesin dimers.

Further experiments with cycled salt concentration indicate that SMC1/3 has two distinct modes of DNA binding: the looping interaction is salt-labile, but at the same time SMC1/3 protein is salt-stable on DNA. This suggests that the hinge forms a salt-stable contact with DNA, while looping is accomplished by weaker salt-labile interactions between the coiled-coil domains and DNA.

### The role of nucleotide binding in DNA compaction

The SMC1/3 heterodimer by itself has slow ATPase activity (20,52), which is stimulated by binding of the Scc1 C-terminus to the SMC3 head (20). Additional experiments with SMC1/3/Scc1-C (Supplementary Text and Supplementary Figure S11) showed that binding of ATP and non-hydrolysable ATP analogs only mildly retard the stepwise compaction reaction. This plus our experiments with the ‘HL’ construct indicate that the DNA compaction by SMC1/3 is not powered by ATP or by its hydrolysis.

It has been observed *in vivo* that ATP hydrolysis appears to play a role in redistribution of cohesin (53). ATP binding may simply switch the complex into a state in which DNA loop-capture binding is suppressed, allowing it to be more easily relocated; ATP hydrolysis then allows rebinding of cohesin to DNA. The ATP-independent DNA compaction we have observed is similar to that seen for the *E.coli* SMC MukBEF, while being precisely the opposite of the ATP-dependent DNA compaction observed for *Xenopus* condensin (19,37,54). However, we do note that cohesin association with chromosomes *in vivo* requires Scc1 (55,56) and ATP binding (14,57,58).

### Cohesin can interact with two pieces of DNA *in trans*

Our braiding experiments show SMC1/3 can bridge two parallel DNAs. It is unclear whether one SMC1/3 dimer can connect two pieces of DNA, or if instead more than one dimer is required for this bridging interaction. Our results provide evidence for a direct physical interaction of cohesin for two pieces of DNA, possibly relevant for its function in mediating sister chromatid cohesion. This direct interaction could facilitate the redistribution of cohesin from inter- to intra-chromatid bridging that occurs at metaphase in yeast (13), possibly without a requirement for reconfiguring cohesin-DNA linkage topology.

In summary, we have observed stepwise structuring of DNA by SMC1/SMC3, which is most simply explained by formation of a series of preferentially positive-writhe loops. The hinge domain is crucial to this mode of binding, while the ATPase head domains play a less crucial role in it. We have also shown that SMC1/3 can act *in trans* to connect two disjoint DNA molecules. ATP bound to SMC1/SMC3/Scc1-C suppresses the DNA folding reaction, suggesting that ATP regulates rather than energetically drives the loop-binding reaction. While our experiments are far from recapitulating *in vivo* cohesin function, our results make clear that SMC1/3 has strong interactions with DNA as well as the ability to organize the double helix into defined structures. We envision direct SMC1/3–DNA interactions as being central to sequestration of cohesin on chromatin and in formation of DNA structures of defined geometry, possibly as a first step preparing cohesin to be topologically ‘locked’ to DNA (15). Our results provide a solid baseline for further experiments with chromatin and interaction partners of SMC1/SMC3/Scc1 aimed at *in vitro* analysis of cohesin’s diverse chromosome-organizing functions.

## SUPPLEMENTARY DATA

Supplementary Data are available at NAR Online: Supplementary Figures 1–11, Supplementary Methods and Supplementary References [59].

Supplementary Data
