# Supplementary material for: The SMC1-SMC3 cohesin heterodimer structures DNA through supercoiling-dependent loop formation
Source: Nucleic Acids Res. 2013 Apr 24;41(12):6149–60. doi: 10.1093/nar/gkt303 (PMC3695518; doi:10.1093/nar/gkt303)
Supplement: Supplementary Data [file supp_41_12_6149__index.html]

The SMC1-SMC3 cohesin heterodimer structures DNA through supercoiling-dependent loop formation — Supplementary Data 

# The SMC1-SMC3 cohesin heterodimer structures DNA through supercoiling-dependent loop formation

## Supplementary Data

files

**Files in this Data Supplement:**

- Supplementary Data - pdf file
