# Supplementary material for: The SMC1-SMC3 cohesin heterodimer structures DNA through supercoiling-dependent loop formation
Source: Nucleic Acids Res. 2013 Apr 24;41(12):6149–60. doi: 10.1093/nar/gkt303 (PMC3695518; doi:10.1093/nar/gkt303)
Supplement: Supplementary Data [file supp_gkt303_revised-SI-130411-for-gkt303.pdf]

Supplementary Information for "The SMC1-SMC3 cohesin heterodimer structures DNA through supercoiling-dependent loop formation"

Supplementary Text

Analysis of kinetics of DNA compaction by SMC1/3

To quantify the effect of supercoiling on DNA compaction by SMC1/3 analysis of experiments using 0.45 pN tension and varied topological states (nicked, ΔLk = -15, 0, +12) was done. Our objective was to take into account the fact that many experiments with ΔLk=0 showed no compaction reaction at all, and to separate the initial lag between protein addition and the start of the compaction reaction from the compaction process itself.

The bandwidth of measurements (rate of measurement of distance between surface bead and tethered bead) was 60 Hz. Data time series shown in the paper show this "raw" data. As mentioned in the methods, most of the dispersion in the data on this time scale is Brownian noise due to tethered bead motion, and not measurement error. For most analysis, the data were replotted on a longer time scale, both with and without moving-box smoothing at 6 Hz (10 data points per smoothed data point). As the typical time between steps visible in the 60 Hz data was in the range of tens of seconds, this rate of smoothing improves resolution of step events (see Fig. S1E).

We considered an experiment to "proceed to compaction" when more than one step was detected over a reaction period of 2 hours. The fraction of reactions that proceeded to compaction in each group is shown in Fig. S2A. Both nicked and positively supercoiled DNA showed 100% folding probability. 70% of the reactions on negatively supercoiled DNA (ΔLk = -15) proceeded to compaction, while ΔLk = 0 DNA had the lowest rate of proceeding to compaction, of about 25%.

For experiments that proceeded to compaction, we measured the compaction fraction (DNA extension after 2 h of reaction as a fraction of the initial extension ($[L_i - L_f]/L_i$ for the trace shown in Fig. 2A, averages shown in Fig. S2B), the lag time (time between protein addition and the first compaction step, T_1-T_0 in Fig. 1C, averages in Fig. S2C), and the reaction rate (rate of extension loss with time from the first step to the point where the final extension was reached, $[L_f$-$L_i]$ / $[T_2$-$T_1]$ in Fig. 2A, averages in Fig. S2D).

The initial extension L_i was determined from tether calibration data acquired before addition of protein to the experiment. The lag time was determined to be the time between addition of protein (T_0, Fig. 2) and the first identifiable step (T_1). The final extension L_f was determined from the terminal region of the time series; the time T_2 at which the final extension was reached was determined to be the time at which the extension first reached within 50 nm of L_f. For extreme cases where there was only one step in the experiment, this amounted to determining T_2 to be the time at which that step occurred. Compaction fraction, lag time, and folding rate showed a marked dependence on DNA topological state. DNA with enough positive torsional stress to reach the corresponding overwinding bucking point (ΔLk = +12 cases of Fig. S2) generated the most robust reaction with a high compaction fraction (Fig. S2B), shortest lag time (Fig. S2C) and the fastest reaction rate (Fig. S2D). For nicked (torsionally unconstrained) molecules, compaction proceeded similarly to those of positive supercoiled DNA. Remarkably, un-nicked, relaxed molecules (ΔLk = 0) not only had the lowest degree of compaction, but also the longest lag time and the slowest reaction rate. Thus ΔLk = 0 was the least favorable topology for compaction by SMC1/3 among the topological states studied. DNA with enough negative torsional stress to reach its buckling point for plectoneme formation (ΔLk = -15) reached about the same compaction fraction as observed for positively supercoiled DNA (≈60%, Fig. S2B), but with a much longer lag time and a slower reaction rate (Fig. S2C-D).

Increasing the amount of plectonemically supercoiled DNA does not enhance the SMC1/3 compaction reaction

Given that positively supercoiled DNAs with ΔLk = +12 led to reliable and fast compaction reactions, we examined the effect of varied levels of positive supercoiling. We note that for the 0.45 pN force used, as ΔLk is increased from 0 to +11, the DNA extension only changes slightly; the ΔLk goes largely into DNA twisting, increasing the torque in the molecule. As ΔLk is increased to values ≥12, plectonemic supercoiling occurs, with almost no change in DNA twisting (constant DNA torque): instead the additional ΔLk increases DNA writhe. Thus by choosing different ΔLk values we could determine whether DNA twist or plectonemic writhe was more important to the compaction reaction.

We carried out experiments for ΔLk = +5, +12, +20 and +30. ΔLk = +5 has about half of the twist of +12, while +20 and +30 have about the same twist as +12 but more plectonemic

crossings (writhe). All these levels of supercoiling led to compaction in every experiment (Fig. S3A). Folding fraction is calculated as a fraction of the extension of plectonemically supercoiled DNA prior to protein addition. Experiments with ΔLk =+12, +20 and +30 had nearly the same compaction fraction, lag time and folding rate (Fig. S3B-D) indicating that increased plectonemic writhe does not accelerate the compaction. However, experiments with ΔLk = +5 showed a lower compcation fraction, a longer lag time, and a slower reaction rate. This indicates that the compaction by SMC1/3 is driven primarily by DNA torsional stress (which increases steadily before plectonemes start to form at ΔLk=+11 (1), rather than by plectonemic interwinding (during plectoneme formation DNA torque is nearly constant).

Nucleotide dependence of DNA folding by the SMC1/3/Scc1-C complex

Previous studies have shown that the Smc1/3 heterodimer alone has very low ATP hydrolysis activity (2,3), therefore we did not pursue studies of ATP binding on SMC1/SMC3-DNA interactions. However, addition of the C-terminal domain of Scc1 (Scc1-C, residue 269-566), which binds to SMC1, is known to stimulate SMC1/3 ATPase activity (3,4). We therefore investigated the effect of varied nucleotides on the DNA compaction reaction driven by the SMC1/3/Scc1-C complex.

DNA compaction activity by the "APO" complex (10 nM SMC1/3-Scc1-C with no ATP added, ΔLk =+12, leftmost bars of Fig. S11A-D) proved similar to that for SMC1/3 (compare to Fig. S2A-D, ΔLk=+12 results). However, including 1 mM ATP in the reaction suppresses the compaction reaction, decreasing the final compaction fraction (Fig. S11B, second bar), slightly boosting the lag time (Fig. S11C) and slowing the reaction rate (Fig. S11D). ADP has a similar compaction-suppressing effect (Fig. S11B-D, third bars). Use of the non-hydrolyzable ATP analog ATPγS generates the strongest suppression of the reaction, reducing the probability of the reaction starting at all (Fig. S11A), reducing the final compaction fraction (Fig. S11B), increasing the lag time (Fig. S11C, fourth bar), and slowing the reaction rate (Fig. S11D).

To further examine the role of ATP occupation at the heads, we also carried out experiments using the wild-type SMC1/3 heterodimer bound to a Scc1-C point mutant (L532R) which has suppressed ATP binding by SMC1 and suppressed hydrolysis of ATP bound to SMC3 (3). The results of using SMC1/3/Scc1-C-L532R+ATP (1 mM ATP, rightmost bars of Fig. S11) were reactions which were intermediate in compaction fraction, lag time, and reaction rate for

SMC1/3/Scc1-C with no ATP (fastest and most compaction) and SMC1/3/Scc1-C+ATPγS (slowest and least compacting). Since for SMC1/3/Scc1-C-L532R+ATP, nucleotide binding occurs at SMC3 but with little or no hydrolysis, while with SMC1/3/Scc1-C+ATPγS, binding but no hydrolysis occurs at both SMC1 and SMC3 (3), we conclude that nucleotide binding tends to suppress both the rate and extent of the DNA-compaction reaction by SMC1/SMC3/Scc1-C.

Two-DNA tethers for braiding experiments

Beads tethered by two or more of pFOS-1(9.6 kb) DNA (nicked, with no torsional stiffness) show a rotation-dependent extension distinct from a single supercoiled DNA, characterized by a shape symmetric around Ca=0, a sharp peak at Ca=0 corresponding to the insertion of the first left- or right-handed crossing (Fig. S9A, B), and a spring constant approximately double that a single DNA (crudely, a requirement of a force of approximately 0.2 pN to achieve a 50% extension of the naked braid) (5-7). Constructs satisfying all of these constraints were accepted as two-molecule tethers.

Following identification of two-molecule tethers and calibration measurements of extension and force as a function of magnet position, Ca was set to -1 to introduce one crossing along the braid, force was set to 0.45 pN, SMC1/3 solution was flowed into the flow cell, and measurements were carried out as for single-DNA tethers.

Supplementary Figures

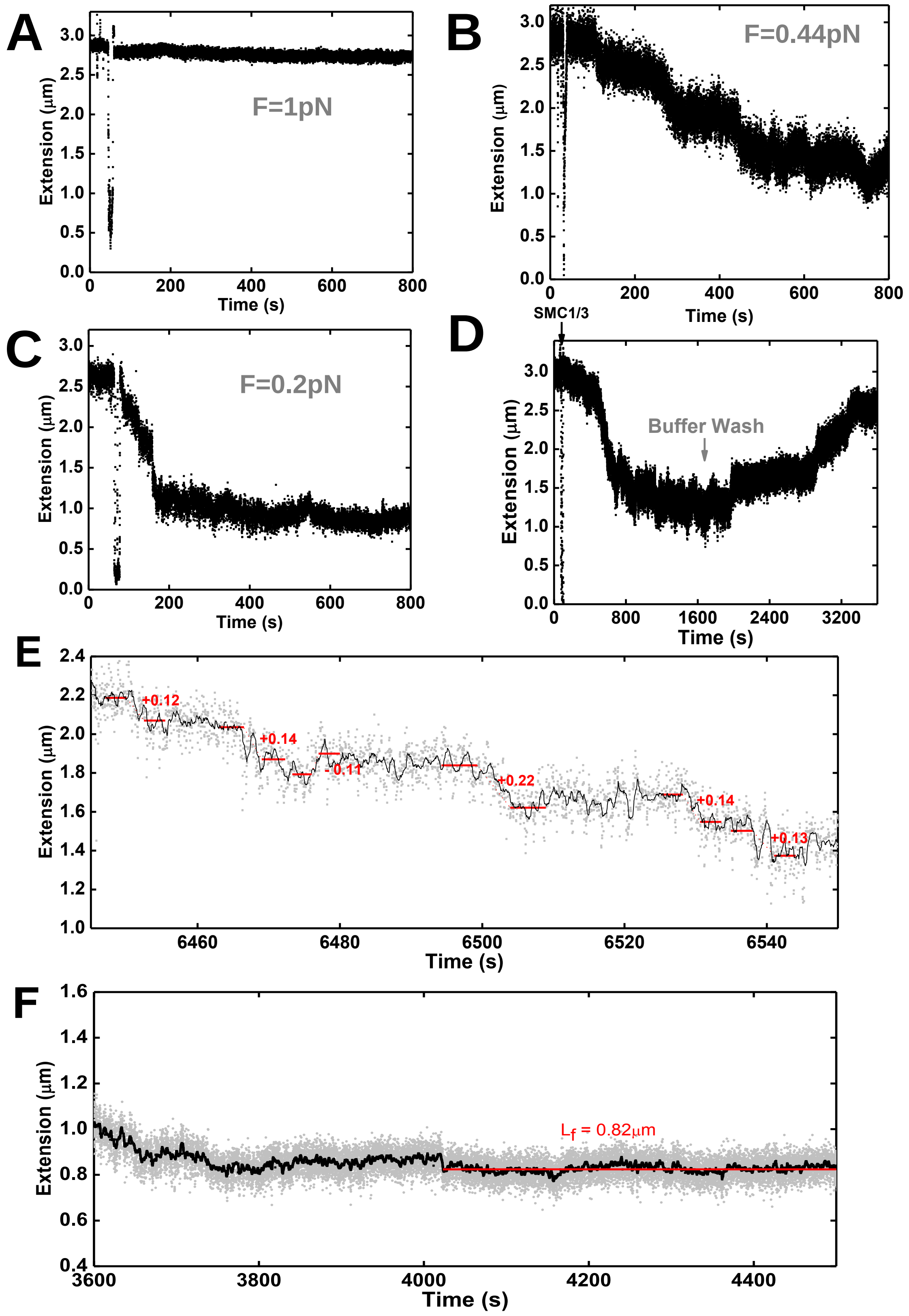

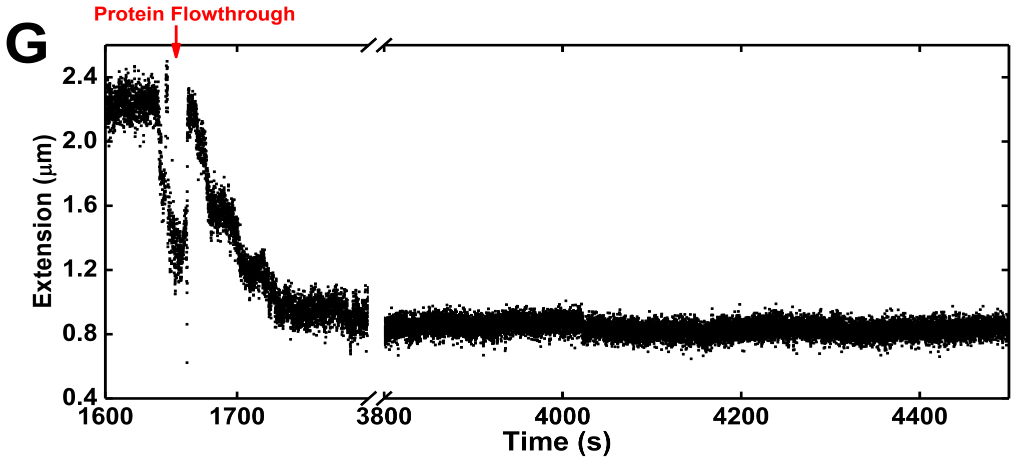

Fig. S1. DNA compaction by SMC1/3 is force dependent. Sample traces of SMC1/3 DNA interaction on nicked DNA under the following applied force: (A) 1.0 pN, (B) 0.44 pN, and (C) 0.2 pN, all with 10 nM of SMC1/3 present.

(D) Unfolding of a tether previously condensed by 10 nM SMC1/3 with ΔLk=+12, following replacement of protein solution with protein-free buffer.

(E) Part of a time series for a ΔLk=+12 compaction run, shown unsmoothed (grey dots) and smoothed (every 10 data points, black line) data, along with steps (red line segments) that were identified along the time series. Step sizes are labeled next to each step in μm.

(F) Tail region of a ΔLk=+12 compaction run, shown unsmoothed (grey dots) and smoothed (every 100 data points, black squares) data, red line indicates the final extension L_f determined from the later part of the time series.

(G) A complete sample trace of a ΔLk=+12 compaction run. Red arrow indicates the time when 10 nM SMC1/3 was introduced. Raw data (black squares) before the break (time=1600-1800) shows realtime DNA compaction after the SMC1/3 protein addition; Data after the break (3800-4500) indicates that final DNA extension stays constant for a long period of time.

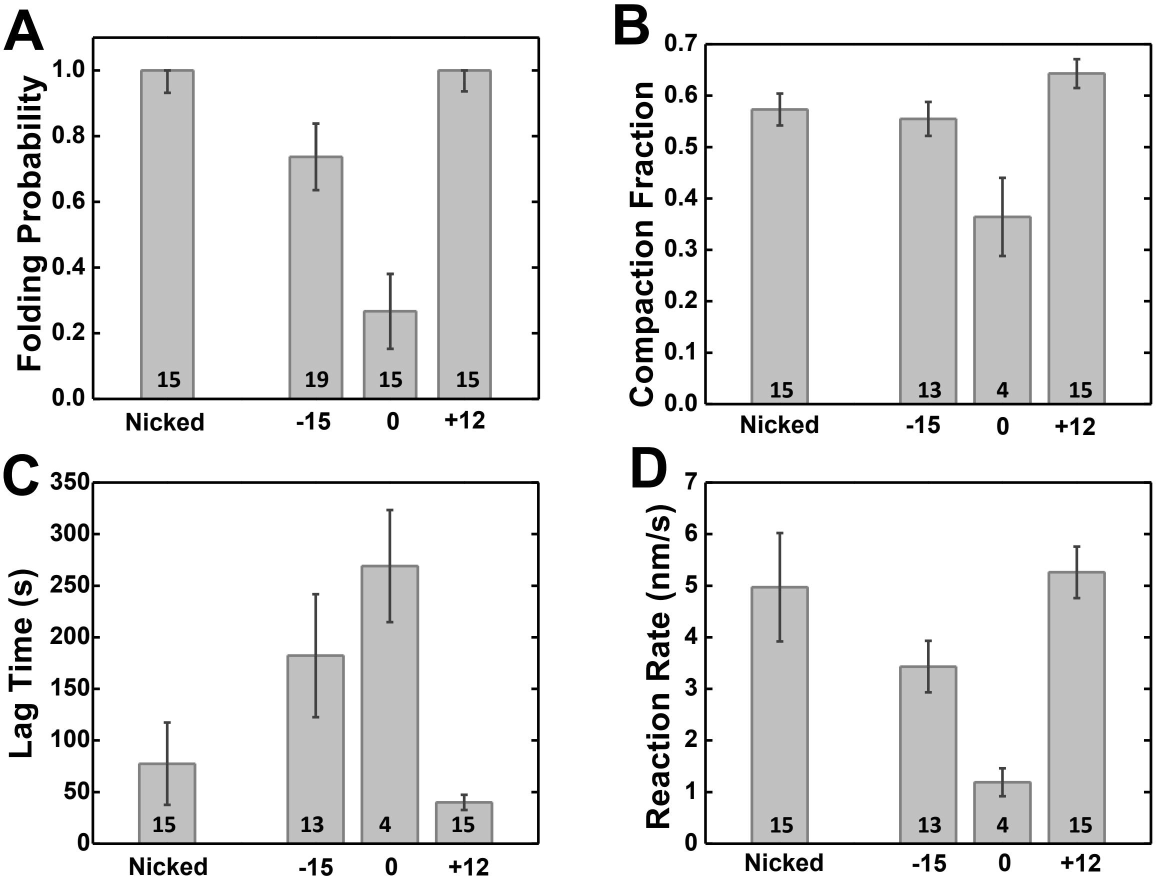

Fig. S2 Supercoiling dependence of kinetics for folding of DNA by cohesin SMC1/3. (A) Folding probability. For each DNA topology, this is the fraction of experiments which proceeded to compaction. Experiments that proceeded to compaction were further analyzed with the following parameters: (B) Compaction fraction measures the final DNA extension after the reaction as a fraction of the initial extension. (C) Lag time measures the time between protein addition and the first detectable compaction step. (D) Reaction rate represents the average rate of extension lost with time from the first step to the point where the final extension was reached. Error bars represent the standard error in each group. For each group of DNA, the number of experiments were done (n) is shown on each bar.

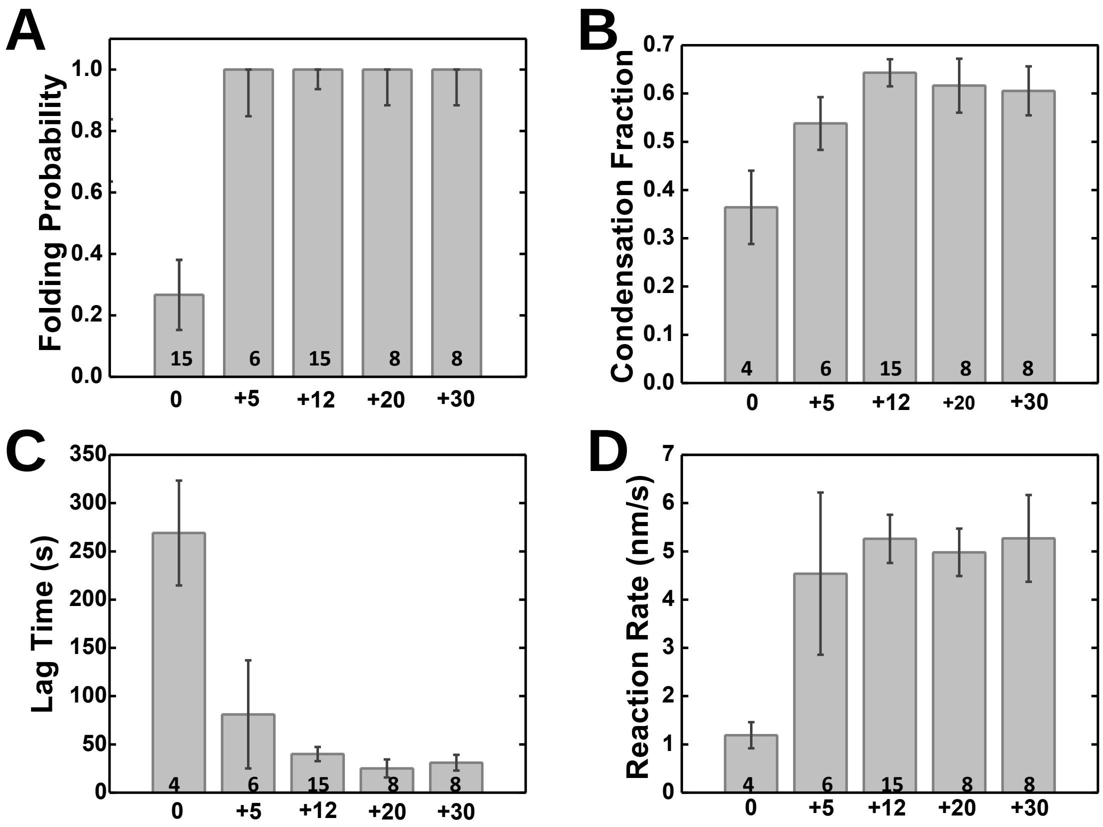

Fig. S3 DNA folding reaction by cohesin SMC1/3 is driven primarily by DNA torsional stress. (A) Folding probability. In each DNA topology group, the percentage of experiments which proceeded to compaction was plotted. Then the experiments that proceeded to compaction were further analyzed with the following parameters: (B) Compaction fraction measures the final DNA extension after the reaction as a fraction of the initial extension of plectonemically supercoiled DNA. (C) Lag time measures the time between protein addition and the first detectable compaction step. (D) Reaction rate represents the average rate of extension lost with time from the first step to the point where the final extension was reached. Error bars represent the standard error in each group. For each group of DNA, the number of experiments were done (n) is labeled on each bar.

.

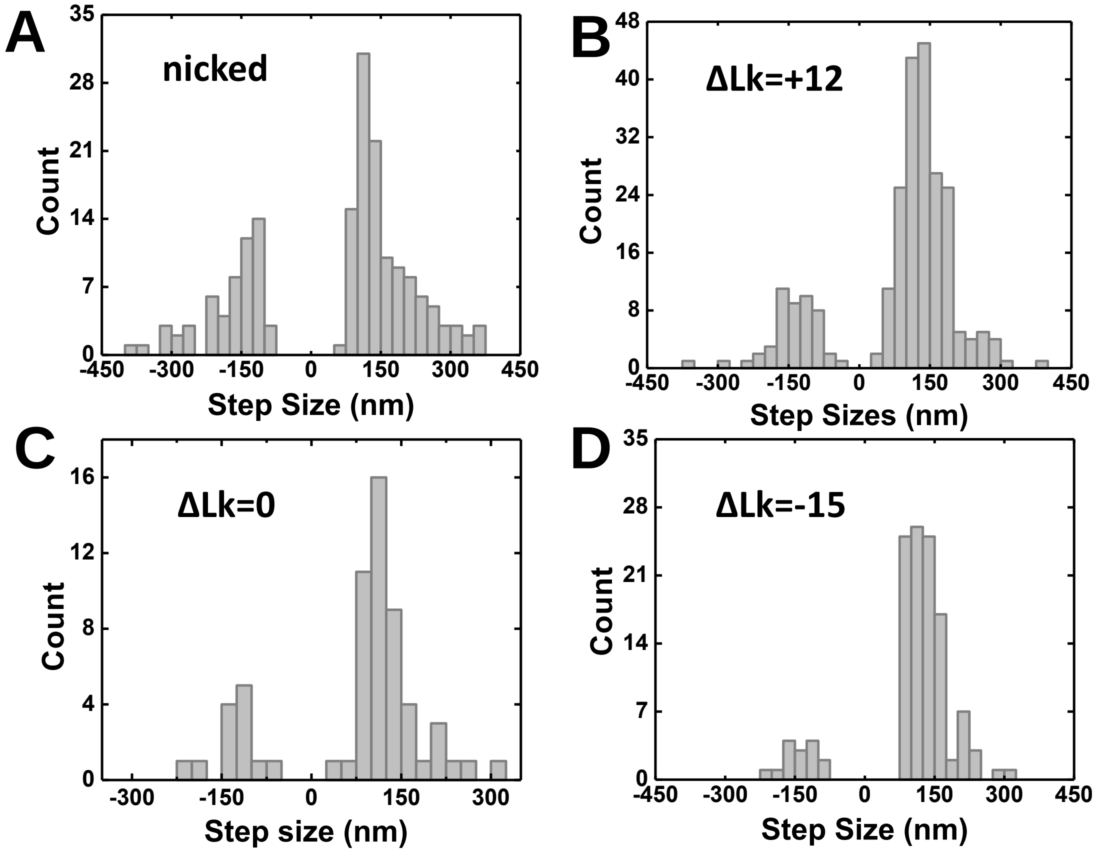

Fig. S4. Step size distribution. (A) nicked DNA, (B) positively supercoiled DNA (ΔLk=+12), (C) un-nicked DNA with ΔLk=0, and (D) negatively supercoiled DNA (ΔLk=-15).

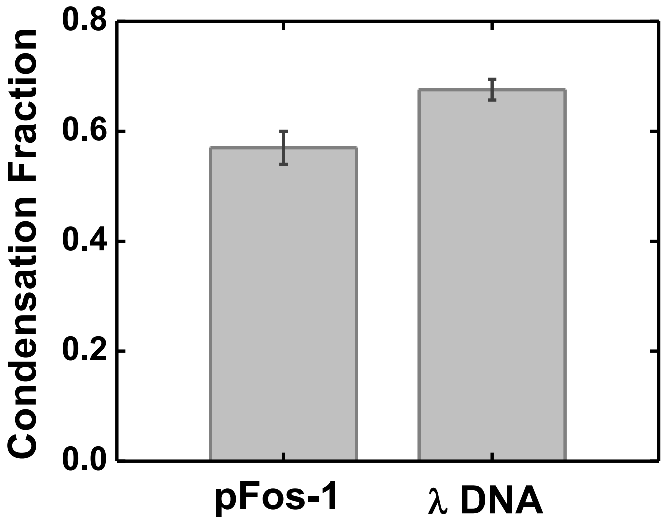

Fig. S5. Compaction fraction on pFos-1 (9.6 kb) versus λ DNA (48.5 kb). Both DNAs were nicked and under 0.45 pN tension. The average compaction fraction for pFos-1 is 0.57 ± 0.03; the average compaction fraction for λ DNA is 0.67 ± 0.02.

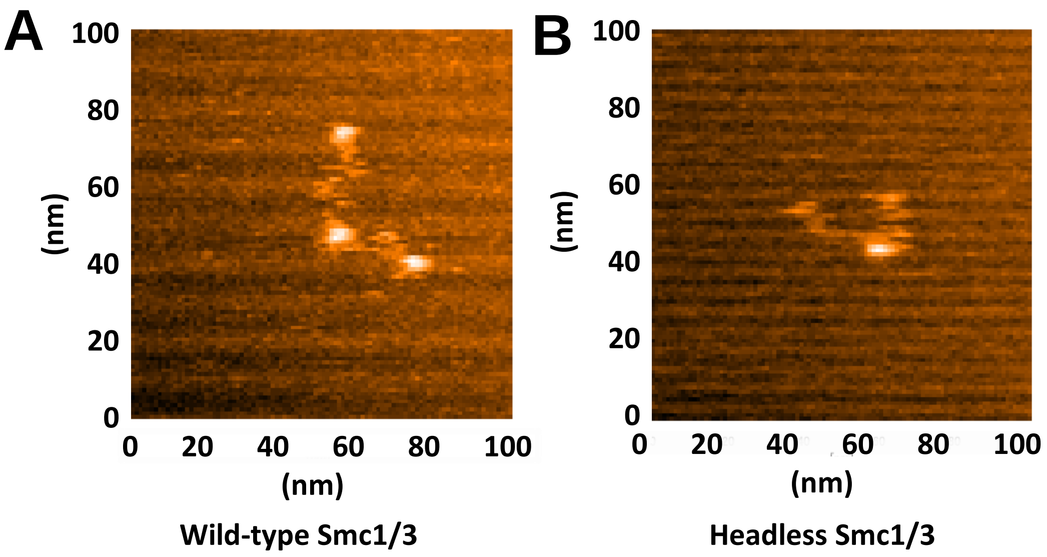

Fig. S6. Images of wild-type SMC1/3 and Headless mutant by Atomic Force Microscopy. (A) High speed AFM image of the SMC1/3 complex visualizes globular domains (ATPase head and hinge domain) and coiled coil regions. Two ATPase domains are on both ends and hinge domain is in the middle. A coiled-coil region tethers the two domains. (B) High speed AFM image of the ATPase headless mutant. Two coiled-coil regions protrude from the hinge domain. This complex lacks the ATPase head domain.

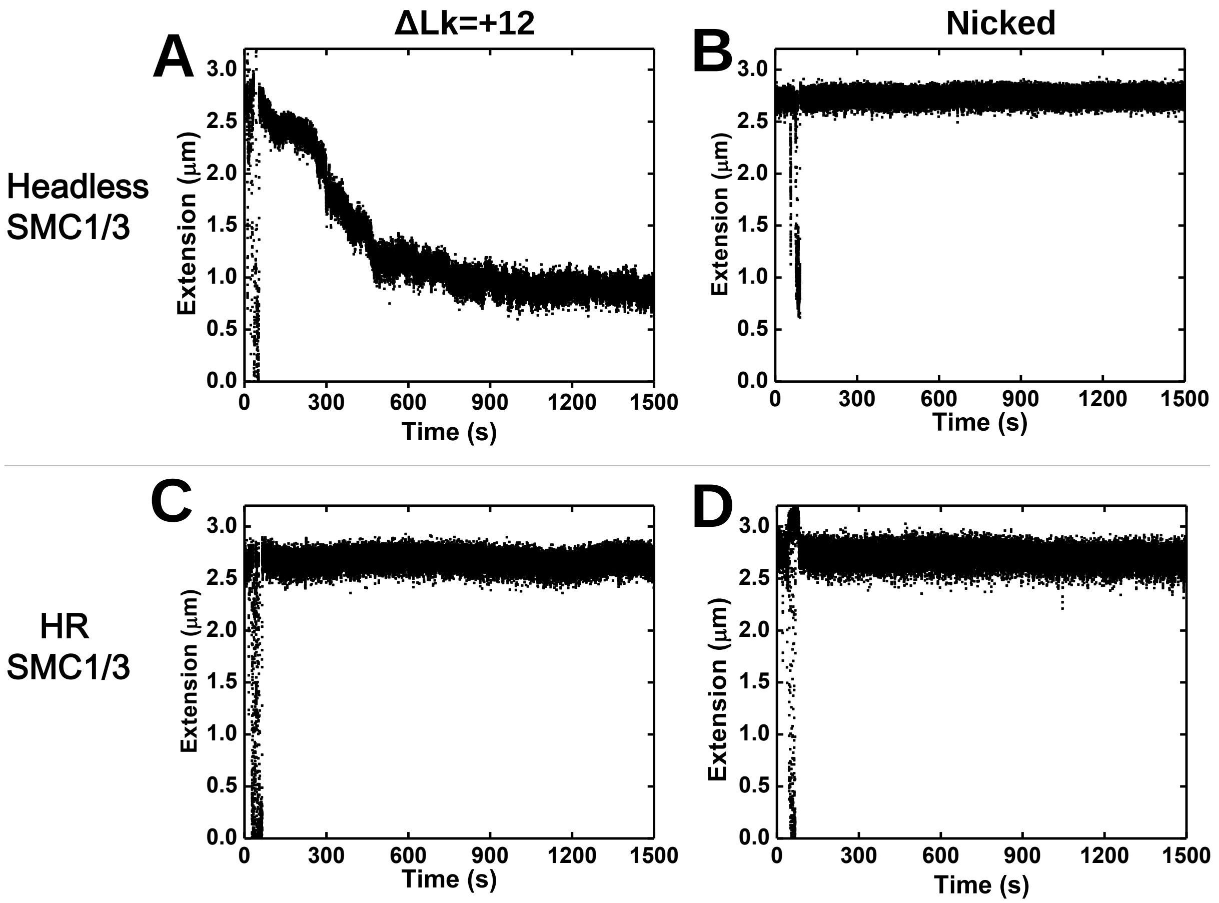

Fig. S7. DNA compaction reaction by SMC1/3 Mutants. (A-B) Sample traces of 80 nM SMC1/3 headless mutant on either DNA with ΔLk=+12 (A), or nicked DNA (B). (C-D) Sample traces of 80 nM hinge-replaced (HR) SMC1/3 on either DNA with ΔLk=+12 (C), or nicked DNA (D). Force was 0.45 pN in all experiments.

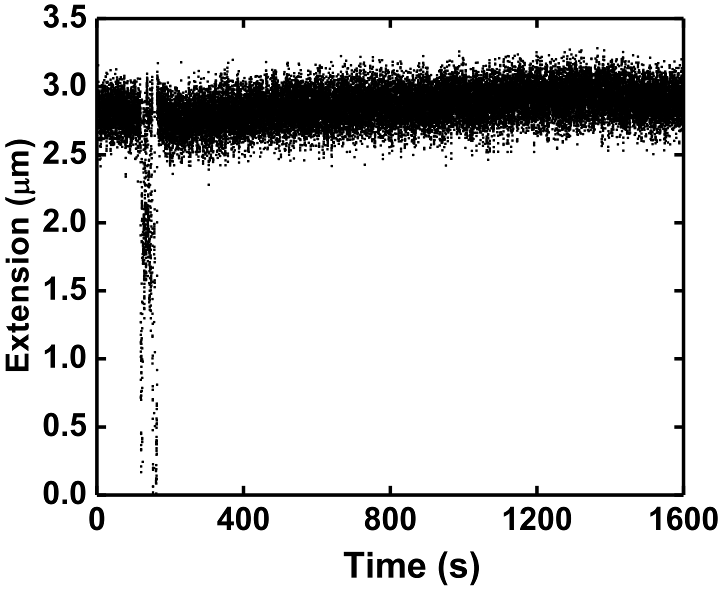

Fig. S8. Hinge alone does not compact DNA. Sample trace for 80 nM hinge domain alone on DNA with ΔLk=+12 (N=7).

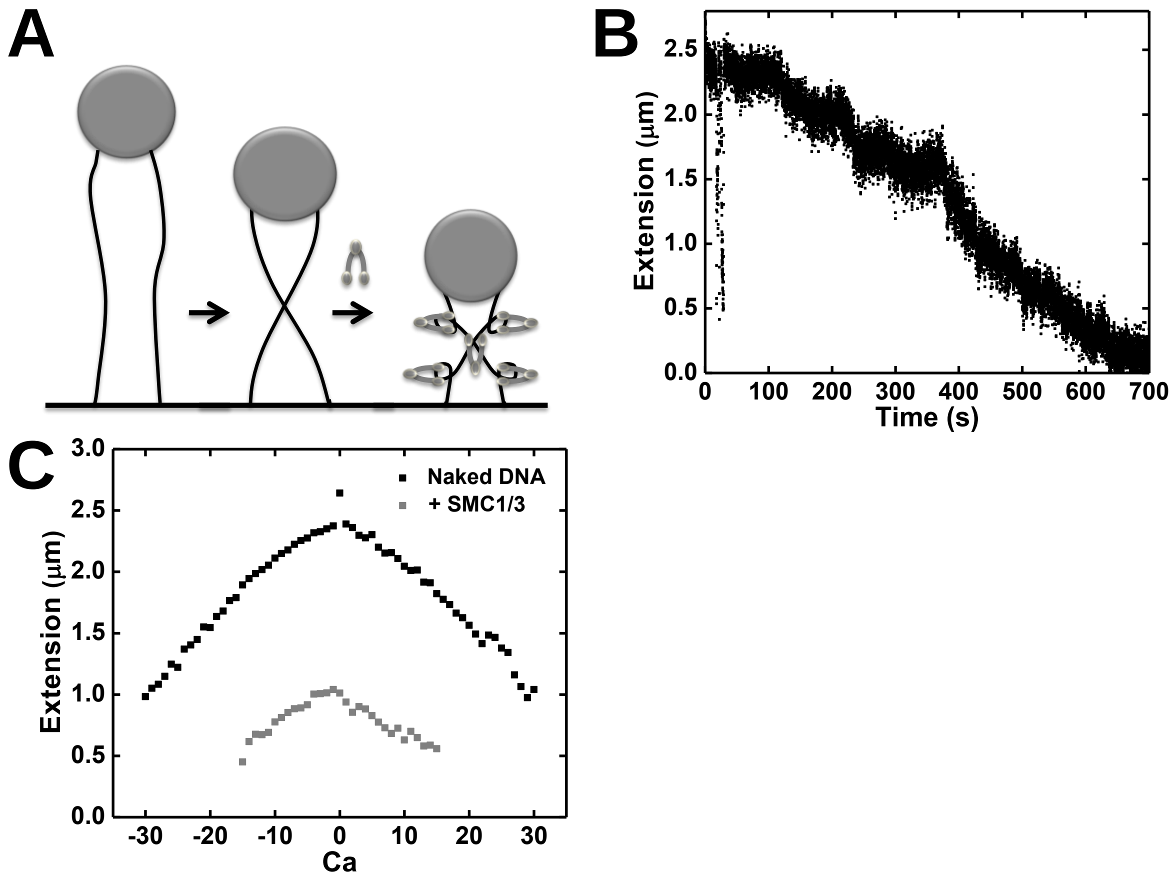

Fig. S9. *In trans* SMC1/3 interaction between braided DNA molecules. (A) Schematic representation of braided DNA. Two 9.8kb nicked DNA molecules tethered between a paramagnetic bead and the cover slip form a two-DNA tether with torsional stiffness. Right-most picture shows two DNA tether at ΔLk=0, after the first turn DNA catenation number of +1 or -1, two DNA molecules make the first crossing, resulting in a sharp drop in tether extension of ~300 nm (panel C, closed squares). Upon protein addition, DNA-protein interaction can be detected both *in cis* and *in trans*. (B) Sample trace of SMC1/3 compacts braided DNA. SMC1/3 was introduced onto braided DNA with catenation of -1, stepwise compaction was observed. At the end of compaction, DNA was almost compacted completely, likely due to both *in cis* and *in trans* interactions. (C) DNA extension versus catenation number for a double DNA tether, for 0.45 pN force, in protein free buffer (black squares) and in 10 nM SMC1/3 (grey squares).

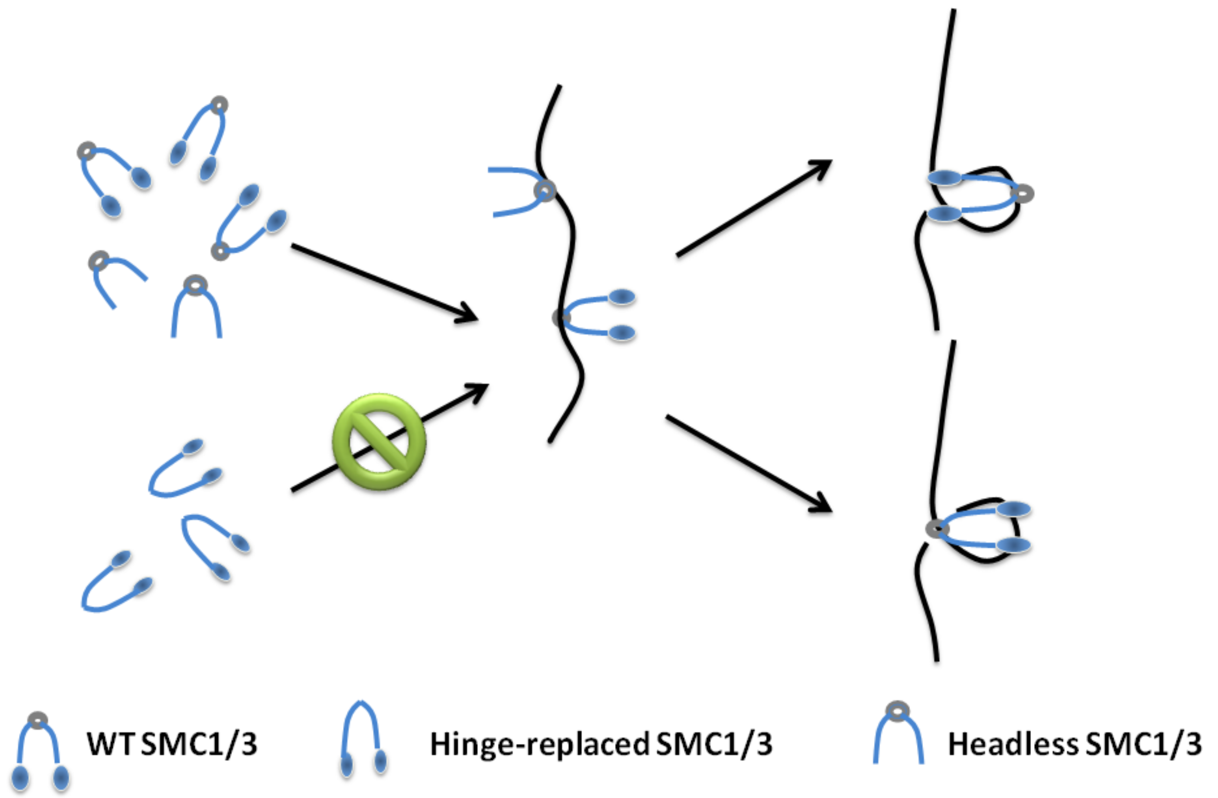

Fig. S10. Proposed mechanism for DNA binding and compaction by cohesin SMC1/3. Hinge domain is critical for DNA binding and compaction. Lack of hinge-DNA contact results in hinge-replaced SMC1/3 failing to compact DNA. After the initial DNA-protein contact, SMC 1/3 can preferentially trap chiral loops along DNA; head domains facilitate loop formation.

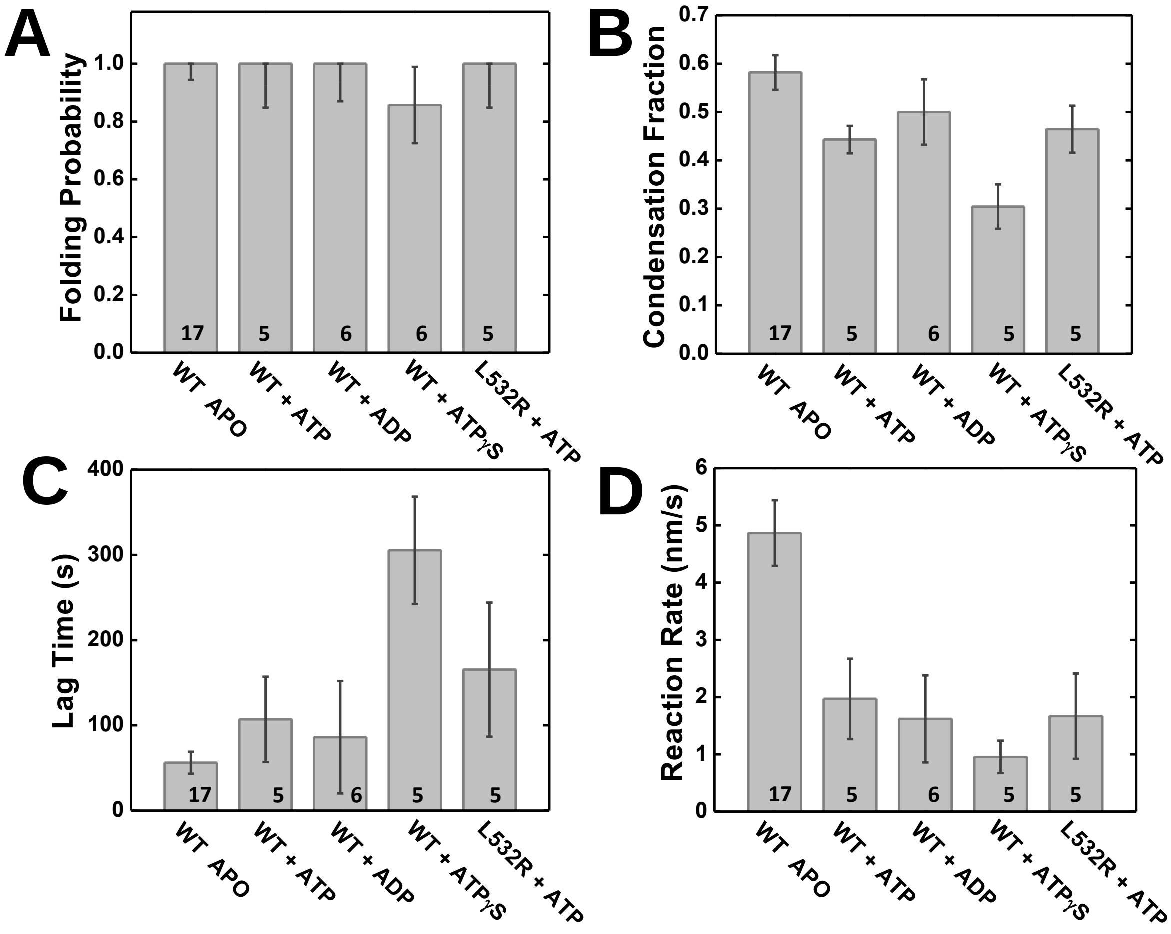

Fig. S11. Nucleotide dependence of DNA folding reaction by cohesin SMC1/SMC3 + Scc1-C terminal domain. (A) Folding probability. The percentage of experiments which proceeded to compaction was plotted. Experiments that proceeded to compaction were analyzed to extract: (B) Compaction fraction, the final DNA extension after the reaction as a fraction of the initial extension; (C) Lag time, the time between protein addition and the first detectable compaction step; (D) Reaction rate, the average rate of extension lost with time from the first step to the point where the final extension was reached. Error bars represent standard error for each group with numbers of trials as indicated.

References

1. Mosconi, F., Allemand, J.F., Bensimon, D. and Croquette, V. (2009) Measurement of the torque on a single stretched and twisted DNA using magnetic tweezers. *Physical review letters*, **102**, 078301.
2. Haering, C.H., Schoffnegger, D., Nishino, T., Helmhart, W., Nasmyth, K. and Lowe, J. (2004) Structure and stability of cohesin's Smc1-kleisin interaction. *Molecular cell*, **15**, 951-964.
3. Arumugam, P., Nishino, T., Haering, C.H., Gruber, S. and Nasmyth, K. (2006) Cohesin's ATPase activity is stimulated by the C-terminal Winged-Helix domain of its kleisin subunit. *Current biology : CB*, **16**, 1998-2008.
4. Gruber, S., Arumugam, P., Katou, Y., Kuglitsch, D., Helmhart, W., Shirahige, K. and Nasmyth, K. (2006) Evidence that loading of cohesin onto chromosomes involves opening of its SMC hinge. *Cell*, **127**, 523-537.
5. Stone, M.D., Bryant, Z., Crisona, N.J., Smith, S.B., Vologodskii, A., Bustamante, C. and Cozzarelli, N.R. (2003) Chirality sensing by Escherichia coli topoisomerase IV and the mechanism of type II topoisomerases. *Proceedings of the National Academy of Sciences of the United States of America*, **100**, 8654-8659.
6. Charvin, G., Bensimon, D. and Croquette, V. (2003) Single-molecule study of DNA unlinking by eukaryotic and prokaryotic type-II topoisomerases. *Proceedings of the National Academy of Sciences of the United States of America*, **100**, 9820-9825.
7. Bai, H., Sun, M., Ghosh, P., Hatfull, G.F., Grindley, N.D. and Marko, J.F. (2011) Single-molecule analysis reveals the molecular bearing mechanism of DNA strand exchange by a serine recombinase. *Proceedings of the National Academy of Sciences of the United States of America*, **108**, 7419-7424.
